# The formation of multi-synaptic connections by the interaction of synaptic and structural plasticity and their functional consequences

**DOI:** 10.1186/1471-2202-15-S1-P186

**Published:** 2014-07-21

**Authors:** Michael Fauth, Florentin Wörgötter, Christian Tetzlaff

**Affiliations:** 1 Third Physics Institute, Bernstein Center for Computational Neuroscience, University Göttingen, 37077, Germany

## 

The connectivity between neurons - i.e., the number of synapses and their transmission efficacies (weights) - determines information processing and storage in neural networks as, for instance, the cortex. Thus, in order to understand the functionality of cortical neural networks, we have to understand how they generate their connectivity. Along this line, there are two major mechanisms to modify connectivity: First, on a timescale of minutes to hours, synaptic plasticity (long-term depression or potentiation) adapts the transmission efficacies (weights) of existing synapses. Second, on a timescale of hours to days, structural plasticity creates and removes synapses. Hereby, the main influence is the volume of the dendritic spine [1] hosting the synapse. This volume is closely linked to the synaptic weight [2], which, in turn, is adapted by synaptic plasticity. All these mechanisms are influenced and determined by the activity pattern of the neural network. Hence, to understand how the connectivity of adult neural networks is adapted or maintained, it is important to understand the interaction of neural activity and the different plasticity mechanisms. In the following we will focus on one property of the cortical connectivity, namely the characteristic, bimodal distributions of the number of synapses connecting two neurons [3-5]. These distributions show high probabilities for two neurons to be either unconnected or connected with multiple (3-8) synapses.

To investigate how these distributions can emerge from the interaction between synaptic and structural plasticity, we use a probabilistic model, which captures the process of structural plasticity by abstracting it with weight-dependent probability functions. This allows us to analytically calculate the distribution of the number of synapses between two neurons and show that the bimodal synapse distributions from cortex will arise when using synaptic plasticity rules with synaptic weights growing with the postsynaptic activity. This is generically fulfilled, for instance, by a combination of Hebb-like plasticity and synaptic scaling [7,8].

Given such a combination, we show that a single set of rules and parameters yields the different biologically observed distributions for different cortical layers of synapses at corresponding activities. However, to guarantee stable connectivity, the distributions should not change spuriously whenever the neural activity varies. Albeit using a probabilistic model, this type of long-term stability surprisingly arises naturally due to a hysteresis which safeguards a connection against overly fast changes. As this hysteresis cannot emerge for connections with single synapse, it can be regarded as a feature of multi-synaptic connections, which may add to the stability of information storage in the network.
